# Synergistic Adsorption–Membrane Distillation for Heavy Metal Extraction and Water Reclamation from Saline Waste Streams

**DOI:** 10.3390/membranes15090271

**Published:** 2025-09-08

**Authors:** Jie Xu, Jinxin Liu, Mei-Ling Liu, Guangze Nie, Dong Zou

**Affiliations:** 1 School of Environmental Science and Engineering, Nanjing Tech University, Nanjing 211816, China; xujie@njtech.edu.cn (J.X.); jinxinliu@njtech.edu.cn (J.L.); 2 NJTECH University Suzhou Future Membrane Technology Innovation Center, Suzhou 215333, China; meilingl@njtech.edu.cn; 3 State Key Laboratory of Materials-Oriented Chemical Engineering, College of Chemical Engineering, Nanjing Tech University, Nanjing 211816, China

**Keywords:** membrane process, Pb(II) removal, water treatment

## Abstract

Membrane distillation demonstrates ideal separation performance towards saline water; however, it fails to accomplish the classification and recovery of multiple components from complex saline solutions (i.e., heavy metal ion-laden saline water in process industries). Herein, an adsorption–membrane distillation (MD) coupling process was proposed, as an example of a Pb(II)/NaCl mixed solution, in which the prepared adsorption membrane was firstly employed to adsorb heavy metal ions in the mixed solution and then the brine was concentrated by the MD process to realize water source recovery and utilization. Firstly, an FeOOH@PVDF adsorptive membrane was fabricated to adsorb Pb(II) ions. It was demonstrated that chemical adsorption was identified as the dominant mechanism, and the composite membrane showed excellent selective adsorption for Pb(II). Following this, the omniphobic membrane was then employed to concentrate the Pb(II)-removed saline solution, maintaining a water flux of 16.12 kg·m^−2^·h^−1^ at a concentration factor of 7.7, demonstrating excellent MD concentration performance. Through this coupled process, the saline wastewater containing heavy metal ions was successfully separated into purified water and concentrated brine without heavy metal ions, providing a novel approach for the treatment and recycling of complex saline wastewater.

## 1. Introduction

Water pollution has emerged as one of the most critical challenges confronting the global community [1,2,3,4]. Among various contaminants, heavy metals represent a particularly significant concern due to their toxicity and potential carcinogenicity. These metals are not only resistant to biodegradation but also exhibit a propensity for bioaccumulation within organisms, leading to progressive concentration magnification along the food chain [5,6]. Heavy metals are defined as toxic metallic elements possessing a specific density exceeding 4.5 g·cm^−3^. Lead (Pb), characterized by its widespread environmental distribution and pronounced biological toxicity [7,8], poses an exceptionally severe threat to both ecological integrity and human health. Intensified resource exploitation activities and the expanding application scope of lead compounds have resulted in the substantial release of lead contaminants into the natural environment. Lead ion-contaminated saline wastewater primarily originates from industrial processes such as mining, battery manufacturing, and leaching from deteriorating infrastructure. Its presence poses significant environmental and public health risks due to the neurotoxicity of lead, which can cause severe physiological disorders and persistent ecological contamination through bioaccumulation.

To remove lead ions from aquatic systems, multiple methods have been developed, including coagulation [9], chemical precipitation [10], biological treatment [11], and adsorption [12]. Among these strategies, adsorption has gained extensive application due to its inherent advantages [13,14]: straightforward design, broad effectiveness against diverse heavy metals, cost-effectiveness, high treatment efficiency, operational simplicity, and proven feasibility for large-scale industrial implementation. Furthermore, adsorbents with tailored properties can be selected to optimize treatment efficacy according to specific application scenarios. Since the performance and efficiency of adsorption are fundamentally governed by the type and characteristics of the adsorbent material, the development of novel adsorbents that are low-cost, highly selective, and possess high adsorption capacities is of paramount importance for the effective mitigation of heavy metal pollution. Iron-based adsorbents [15,16], characterized by their abundant reserves, low cost, and environmentally benign nature, are regarded as ideal materials for the removal of toxic heavy metal ions from aqueous solutions. Their high efficiency, reactivity, and superior adsorption capacity offer novel solutions for water purification. Among various iron-based nanomaterials, iron oxyhydroxide (FeOOH) stands out due to its unique physicochemical properties. This material exhibits exceptional stability under acidic conditions, conferring a significant advantage for treating acidic wastewater. Its high specific surface area provides abundant adsorption sites for heavy metal ions. Furthermore, the hydroxyl and iron-oxo groups on the FeOOH surface confer strong binding affinity, enabling the formation of stable chemical bonds with diverse organic and inorganic substances [17,18]. This characteristic facilitates the efficient removal of heavy metal ions such as Pb(II) from water.

However, nano-sized nanoparticles are prone to agglomeration during adsorption processes due to their high specific surface area. This agglomeration diminishes their contact area with heavy metal ions in aqueous solutions, consequently impairing adsorption efficacy. Furthermore, nanoparticle adsorbents present significant challenges for recovery and reuse post adsorption. Recent studies have demonstrated that synergistic integration of nano-adsorbents with membrane materials yields substantially enhanced adsorption performance [19,20]. In our previous work [21], we developed a MoS_2_-PAN hybrid composite membrane by blending MoS_2_ nanoparticles with a polyacrylonitrile (PAN) matrix. This composite membrane exhibited exceptional adsorption capacity for chromium (Cr) ions in aqueous systems. Moreover, the adsorption process demonstrates considerable potential for integration with membrane distillation (MD) for treating complex industrial wastewater. For example, Aftab et al. [22] explored a process integrating pretreatment via adsorption using biomass charcoal and activated carbon with membrane distillation for landfill leachate treatment. This integrated approach significantly mitigated membrane fouling, enhanced membrane flux, and extended membrane lifespan. Enhancing the performance of such integrated systems hinges on two critical aspects: firstly, the development of adsorbent materials possessing high adsorption capacity and selectivity to further improve pollutant removal efficacy from wastewater; and secondly, the selection of membrane materials exhibiting high mechanical strength, hydrophobicity, fouling resistance, and anti-wetting properties to optimize the long-term operational stability of the MD process [23,24,25,26].

**Figure 1:**
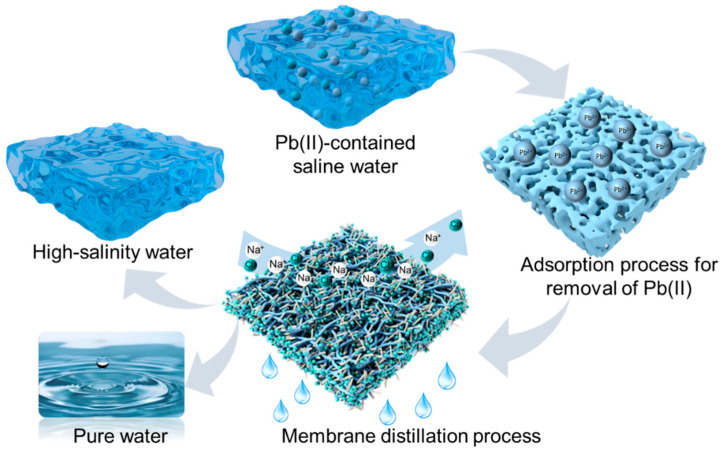
Schematic diagram of the adsorption–membrane distillation coupled process for the treatment of Pb(II)-containing saline water.

This work proposes an innovative strategy combining adsorption technology with the membrane distillation process, aimed at developing an integrated process for the efficient treatment of saline wastewater containing heavy metal ions (as illustrated in Figure 1). Initially, an FeOOH@PVDF adsorptive membrane was fabricated via the phase inversion method. Investigations were conducted into the influence of the FeOOH-to-PVDF ratio on membrane properties, with the aim of optimizing the overall membrane performance for the effective removal of target Pb(II) ions from the solution. Subsequently, the treated solution was concentrated using an MD system. The omniphobic membrane, prepared in our previous work [27], was employed for the concentration and separation of the pre-treated saline wastewater. Through this comprehensive treatment process, the saline wastewater containing Pb(II) ions was successfully separated into purified water and concentrated brine without Pb(II). This approach not only achieved wastewater desalination but also facilitated the subsequent resource recovery of the wastewater and the reclamation of heavy metal ions.

## 2. Experimental

### 2.1. Materials

Polyvinylidene fluoride (PVDF) powder (Solef 6010, Mw: ~573,000) was provided from Solvay Specialty Polymers (Bollate, Italy) and dried at 333 K for at least 4 h to eliminate the absorbed water. Fe(NO_3_)_3_·9H_2_O, polyvinylpyrrolidone (PVP, K30), NaCl, Na_2_SO_4_, NaNO_3_, NaOH, HCl, and Pb(NO_3_)_2_ were provided by Sinopharm Chemical Reagent (Beijing, China). N, N-Dimethylformamide (DMF) was purchased from the Aladdin Company (Shanghai, China). The reagents were used without further purification. A stock solution of 1000 mg∙L^−1^ Pb was prepared by dissolving Pb(NO_3_)_2_ in deionized water. Deionized water was used in the whole experiment. The solution was prepared by dissolving Pb(NO_3_)_2_ and NaCl in deionized water to simulate industrial saline wastewater containing Pb(II). The initial concentrations of Pb(II) and NaCl were 10 mg/L and 3.5 wt% in the synthetic wastewater.

### 2.2. Synthesis of FeOOH Nanopowders

A 2.0 mmol portion of FeCl_3_·6H_2_O was dissolved in 40.0 mL deionized water. The solution pH was adjusted to ~12.0 by dropwise addition of 2.0 mol·L^−1^ NaOH under constant magnetic stirring. After 30 min of continued stirring to complete the reaction [17], the precipitate underwent repeated deionized water washes until the supernatant reached pH~7.0. Vacuum filtration was then performed, followed by drying at 60 °C for 12 h. The dried product was finally ground to a fine powder for further use.

### 2.3. Fabrication of FeOOH@PVDF Membrane

FeOOH@PVDF adsorptive membranes were fabricated using the phase inversion method. The detailed procedure was as follows: A predetermined mass of FeOOH powders (specific ratios are listed in Table 1) was added to 16.50 g of N,N-dimethylformamide (DMF) and magnetically stirred at 60 °C until uniformly dispersed. Subsequently, a measured quantity of polyvinylidene fluoride (PVDF) powder was slowly added, and stirring continued for 6 h to achieve complete dissolution, forming a homogeneous casting solution. The casting solution was then uniformly coated onto a clean glass plate at room temperature using a casting knife (purchased from Elcometer Ltd. (Manchester, UK)), with the thickness of ~200 µm. The glass plate was immediately immersed in deionized water to initiate the phase inversion process. Finally, the formed membrane was immersed in deionized water overnight to thoroughly remove the residual solvent, followed by drying at room temperature for subsequent use [18].

### 2.4. Characterization of the Adsorption Membrane

The morphologies of the samples were examined using scanning electron microscopy (SEM, SU8020, Hitachi, Tokyo, Japan). X-ray diffraction analysis was conducted by an X-ray diffractometer (RIGAKU MiniFlex600, Rigaku, Tokyo, Japan). The tensile strength of the membrane was measured using a tensile testing machine (AGS-J 500N, Shimadzu, Kyoto, Japan). The microstructure and morphology of the synthesized FeOOH nanopowders were characterized by transmission electron microscopy (TEM, FEI Talos F200X, Hillsboro, OR, USA). Energy-dispersive X-ray spectroscopy (FESEM S4800, Hitachi, Tokyo, Japan) was employed to analyze the elemental composition of the membrane surface.

### 2.5. Adsorption Characterization of the Simulated Waste Water

The concentration of Pb and Fe in the solution sample was quantified using inductively coupled plasma (ICP) emission spectroscopy analysis (ICP-OES, ICP-5000, Focused Photonics Inc., Hangzhou, China).Batch adsorption experiments were performed by adding 10 mg of the FeOOH@PVDF membrane (cut into small pieces) to 50 mL of Pb(II) solution, agitated at 140 rpm for 24 h in a constant-temperature shaker. A series of tests were conducted at an initial Pb(II) concentration of 10 mg·L^−1^ to assess the effectiveness of the FeOOH@PVDF membranes in adsorbing Pb(II), examining the influence of key parameters such as iron content in the FeOOH@PVDF membranes, pH, contact duration, and competing ions. In the adsorption experiments, dilute HCl and NaOH solutions of negligible volume were used to adjust the solution pH to predetermined values. The effect of common coexisting cations (Na^+^, Cd^2+^, Ni^2+^, Cu^2+^, Zn^2+^) was studied using their nitrate salts. To investigate the adsorption kinetics, samples were collected and analyzed at different time points by adding 25 mg of AM3 into 500 mL of a 25 mg·L^−1^ Pb(II) solution.

### 2.6. MD Characterization of the Simulated Waste Water

A lab-scale vacuum membrane distillation (VMD) setup was utilized to concentrate the saline water after removing Pb(II). First, the flat sheet membranes were cut into a circle shape with a diameter of 47 mm and then placed into a membrane module. The cell area was about 17.3 cm^2^. The feed solution was the saline water after removing the Pb(II) by the adsorption membrane. The feed solution was circulated at a flow rate of 200 mL/min by peristaltic pumps. After that, the feed solution and vacuum pressure on the condensation side were controlled at 70 °C and 0.0970 MPa. The water flux was calculated using Equation (1). The conductivity of the feed solution and condensate water was measured to gain the rejection of the saline water system by Equation (2).

(1) $$J=\frac{∆m}{A}\cdot t$$

(2) $$R=\left(1-\frac{Cp}{Cf}\right)$$

where Δ_m_ (kg) represents the mass of condensate water in a certain time interval (t, h), and A refers to the effective membrane area (m^2^). *C_P_* and *C_F_* represent the salt concentrations of the permeate and feed solution, respectively.

## 3. Results and Discussion

### 3.1. Characterization of FeOOH Powders and Membranes

**Figure 2:**
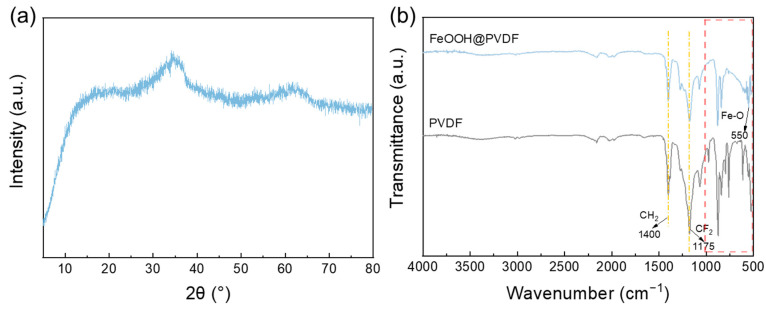

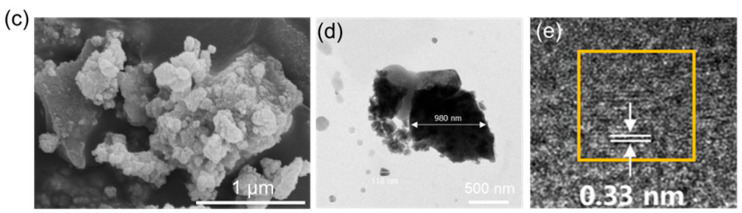
(**a**) XRD pattern of FeOOH, (**b**) ATR-FTIR spectrum of PVDF membrane and FeOOH@PVDF membrane, (**c**) SEM, (**d**) TEM, and (**e**) HRTEM of FeOOH nanoparticles.

The XRD pattern of the as-prepared FeOOH (Figure 2a) shows two broad and weak peaks with no sharp diffraction peaks, indicating an amorphous structure [28]. This can be attributed to the low drying temperature during the synthesis process, which inhibited crystallization. As shown in the ATR-FTIR spectra (Figure 2b), the FeOOH@PVDF adsorption membrane displayed a prominent characteristic absorption peak at 550 cm^−1^, which corresponds to the stretching vibration of the Fe-O bond. This result confirmed the successful incorporation of FeOOH within the PVDF matrix. Additionally, the characteristic peaks observed at 1400 cm^−1^ and 1175 cm^−1^ were attributed to the stretching vibrations of CH_2_ and CF_2_ groups in PVDF polymers [19].

SEM analysis revealed that the FeOOH nanoparticles possessed an irregular polyhedral structure with a rough surface. This morphological characteristic was advantageous for increasing the specific surface area and providing abundant active sites. The TEM and HRTEM images confirmed the nanoscale dimensions and amorphous nature of FeOOH, consistent with the XRD findings. However, locally ordered lattice fringes were observable in certain regions with an interplanar spacing of 0.263 nm, corresponding to the (110) plane of the crystalline FeOOH.

### 3.2. Adsorption Process in the Pb(II)-Contained Saline Water

#### 3.2.1. Effect of Solution pH

**Figure 3:**
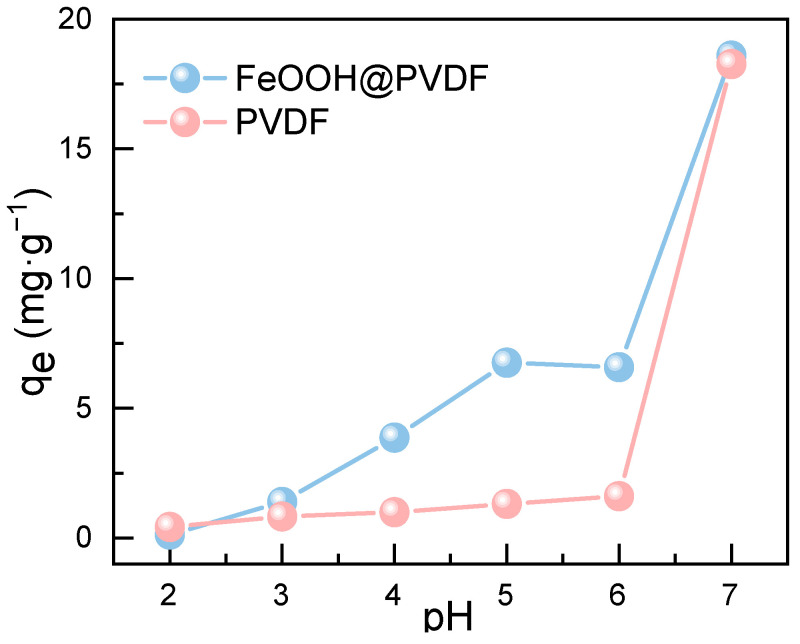
Effect of pH on the adsorption performance of PVDF and FeOOH@PVDF membrane for Pb(II).

To investigate the effect of pH on the adsorption behavior of Pb(II), the differences in adsorption performance of the resulting FeOOH@PVDF adsorption membrane(with AM1 as an example) and the pristine PVDF membrane towards Pb(II) ions in solutions with varying initial pH values (ranging from 2.0 to 7.0) were examined. As illustrated in Figure 3, the equilibrium adsorption capacity of the FeOOH@PVDF membrane exhibited an increasing trend with rising solution pH. Under lower pH conditions, the abundant H^+^ ions present in the solution competed with Pb(II) ions for adsorption sites. Concurrently, the presence of H^+^ ions induced the protonation of surface -OH functional groups on the membrane, rendering them positively charged. This positive charge generated an electrostatic repulsion towards the similarly charged Pb(II) ions, thereby hindering their contact and binding with the adsorptive membrane and consequently reducing the adsorption capacity [20]. As the pH increased, the surface charge state of the adsorptive membrane underwent changes, and the surface functional groups experienced deprotonation. These alterations enhanced the adsorption capability of the membrane towards metal ions [21]. However, when the solution pH further increased to 7.0, Pb(II) ions underwent hydrolysis and formed hydroxide precipitates, predominantly as Pb(OH)_2_ [22]. Therefore, the optimal adsorption pH for Pb(II) in this work was determined to be 6.0. In contrast, the adsorption capacity of the pristine PVDF membrane for Pb(II) ions was negligible. This result clearly demonstrated that the FeOOH nanoparticles predominantly governed the adsorption process. Although a certain degree of “adsorption” effect was observed for the PVDF membrane material at pH 7.0, this was primarily attributable to the hydrolysis and precipitation of Pb(II) ions occurring at this pH, rather than any intrinsic adsorption capability of the PVDF membrane itself.

#### 3.2.2. Effect of FeOOH Content 

**Figure 4:**
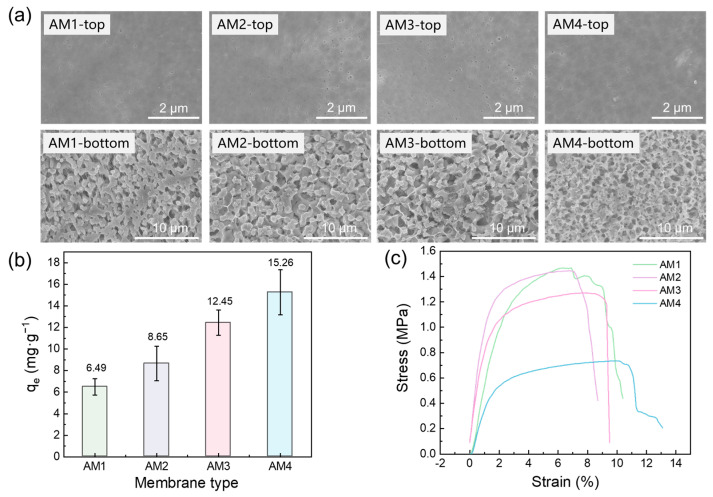
(**a**) SEM images, (**b**) Pb(II) adsorption capacity, and (**c**) stress–strain curves of AM1, AM2, AM3, and AM4 membranes.

The microscopic morphological characteristics and Pb(II) equilibrium adsorption capacities of FeOOH@PVDF adsorption membranes prepared under different parameters are presented in Figure 4. As shown in Figure 4a, all membranes fabricated via the phase inversion method exhibited a typical asymmetric structure, featuring a relatively dense surface layer and a sponge-like pore structure in the bottom layer. This configuration contributed to the enhanced mechanical strength of the membrane and facilitated the adsorption process. Figure 4b reveals that the equilibrium adsorption capacity for Pb(II) increases progressively with higher FeOOH content. The adsorption capacities for membranes AM1, AM2, AM3, and AM4 were 6.49 mg·g^−1^, 8.65 mg·g^−1^, 12.45 mg·g^−1^, and 15.26 mg·g^−1^, respectively. This enhancement in adsorption capacity was primarily attributed to the increased number of adsorption sites provided by the incorporated FeOOH nanoparticles. However, during the preparation of membrane AM4, the insufficient PVDF content resulted in difficulties in membrane formation and pronounced surface heterogeneity in the resulting composite membrane. This heterogeneity was detrimental to the mechanical properties of the membranes as evidenced by its significantly lower tensile strength compared to AM3 (See Figure 4c). Therefore, considering the feasibility of membrane preparation, adsorption performance, and structural integrity, the AM3 membrane was selected as the optimal adsorbent material.

**Figure 5:**
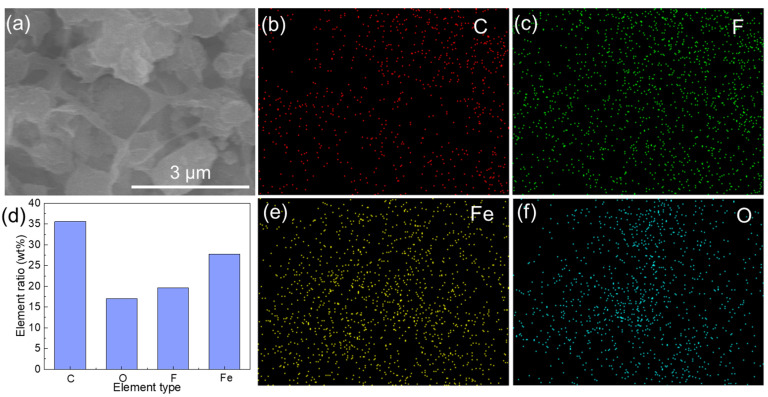
The elemental characterization results of the AM3 membrane. (**a**) SEM image of the membrane bottom surface, (**b**) C, (**c**) F, (**d**) elemental composition table, (**e**) Fe, and (**f**) O.

EDX was subsequently employed to analyze the elemental composition of the AM3 adsorptive membrane, and the results are displayed in Figure 5. The spectrum confirmed the presence of four characteristic elements, C, O, F, and Fe, with atomic percentages of 35.63%, 17.01%, 19.62%, and 27.74%, respectively. The C and F elements originated from the PVDF powder, while the Fe and O elements were derived from the FeOOH powders. In addition, these elements were well-distributed on the membrane surface, indicating that the HFO nanoparticles were well dispersed in the matrix of the PVDF membranes. The other properties of the AM3 membrane are provided in Table S1.

#### 3.2.3. Effect of Adsorption Time

**Figure 6:**
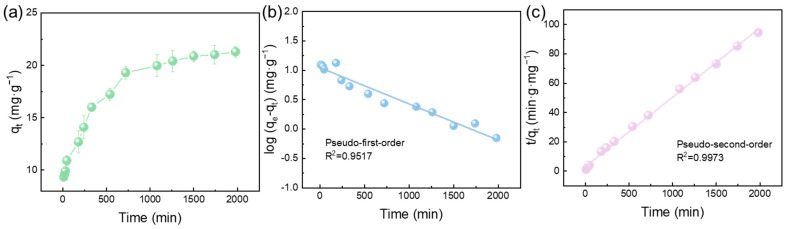
(**a**) Kinetic adsorption profile of Pb(II) on FeOOH@PVDF membrane, with fitting results for (**b**) pseudo-first-order and (**c**) pseudo-second-order models.

The adsorption kinetics curve of Pb(II) ions onto the FeOOH@PVDF membrane is presented in Figure 6a. It can be seen that the membrane exhibited a rapid adsorption rate for Pb(II) within the initial 720 min, followed by a gradual decrease in adsorption velocity until adsorption equilibrium was attained at 1500 min. This characteristic adsorption kinetic profile can be attributed to the following mechanisms: Initially, the rapid adsorption rate arises from the high concentration of metal ions in solution and abundant active binding sites on the membrane surface. Both factors synergistically promoted effective binding between the metal ions and the membrane. As adsorption proceeds, declining metal ion concentrations coincide with reduced availability of binding sites. This diminished concentration gradient substantially restricts further diffusion of ions toward the membrane surface, progressively lowering the adsorption rate until equilibrium is attained.

To investigate adsorption kinetics, experimental data were modeled using pseudo-first-order (Equation (3)) and pseudo-second-order (Equation (4)) kinetic equations:

(3) $$\log\left(q_{e}-q_{t}\right)=\log q_{e}-\frac{k_{1}}{2.303}t$$

(4) $$\frac{t}{q_{t}}=\frac{1}{k_{2}q_{e}^{2}}+\frac{1}{q_{e}}t$$

where q_e_ and q_t_ represent adsorption capacities (mg·g^−1^) at equilibrium and time t, respectively, while k_1_ (min^−1^) and k_2_ (g·mg^−1^·min^−1^) denote corresponding rate constants. Table 2 revealed significantly higher correlation coefficients (R^2^) for the pseudo-second-order model versus the pseudo-first-order model. This superior fit indicated that adsorption kinetics follow pseudo-second-order behavior, implying predominant chemisorption through chemical bonding between metal ions and membrane functional groups.

#### 3.2.4. Effect of Initial Pb(II) Concentrations

**Figure 7:**
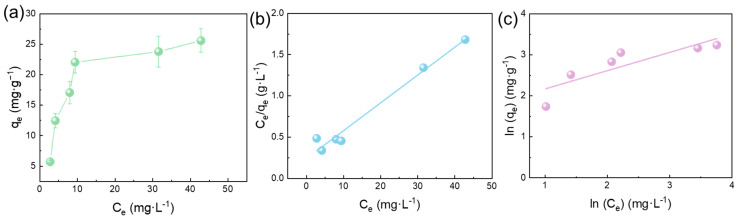
(**a**) Effect of adsorbate concentration on the Pb(II) adsorption by FeOOH@PVDF membrane; equilibrium isotherm of FeOOH@PVDF membrane for Pb(II) ion adsorption by the (**b**) Langmuir and (**c**) Freundlich isotherm models.

The isothermal adsorption data is crucial for describing the adsorption equilibrium state and thermodynamic information, providing key insights into the adsorption mechanism. To construct the adsorption isotherm, experiments were conducted at varying initial Pb(II) concentrations, and the corresponding equilibrium concentrations (Cₑ) and adsorption capacities (Qₑ) were then calculated and plotted. As illustrated in Figure 7a, the adsorption isotherm of Pb(II) onto the FeOOH@PVDF membrane demonstrated that the equilibrium adsorption capacity initially increased rapidly with rising initial metal ion concentration before plateauing. Within the low-concentration range, the equilibrium adsorption capacity increased sharply with increasing initial Pb(II) concentration. However, as the initial concentration further increased, the adsorption capacity approached a saturation value. This occurred primarily because adsorption took place predominantly on the membrane surface; after reaching a certain adsorption level, the capacity stabilized at its maximum. To investigate the adsorption isotherm mechanism, experimental data were fitted with Langmuir and Freundlich models. The Langmuir equation (Equation (5)) characterized monolayer adsorption on homogeneous surfaces with finite identical sites:

(5) $$\frac{C_{e}}{q_{e}}=\frac{C_{e}}{q_{m}}+\frac{1}{q_{m}K_{L}}$$

where C_e_ (mg·L^−1^) and q_e_ (mg·g^−1^) denote equilibrium ion concentration and adsorption capacity, respectively; q_m_ (mg·g^−1^) is the maximum adsorption capacity; and K_L_ (L·mg^−1^) is the Langmuir adsorption equilibrium constant.

Conversely, the Freundlich model (Equation (6)) described multilayer physical adsorption on heterogeneous surfaces:

(6) $$\ln q_{e}=\ln K_{F}+\frac{\ln C_{e}}{n}$$

where K_F_ represents the Freundlich constant [(mg·g^−1^)(L·mg^−1^)1/n], and n indicates adsorption intensity. As summarized in Table 3, the Langmuir model yielded a higher correlation coefficient (R^2^ = 0.9765) than the Freundlich model (R^2^ = 0.7448). This superior fit implied monolayer chemisorption of Pb(II) on the membrane, occurring via chemical bonding at finite active sites.

#### 3.2.5. Effect of Adsorption Temperature

**Figure 8:**
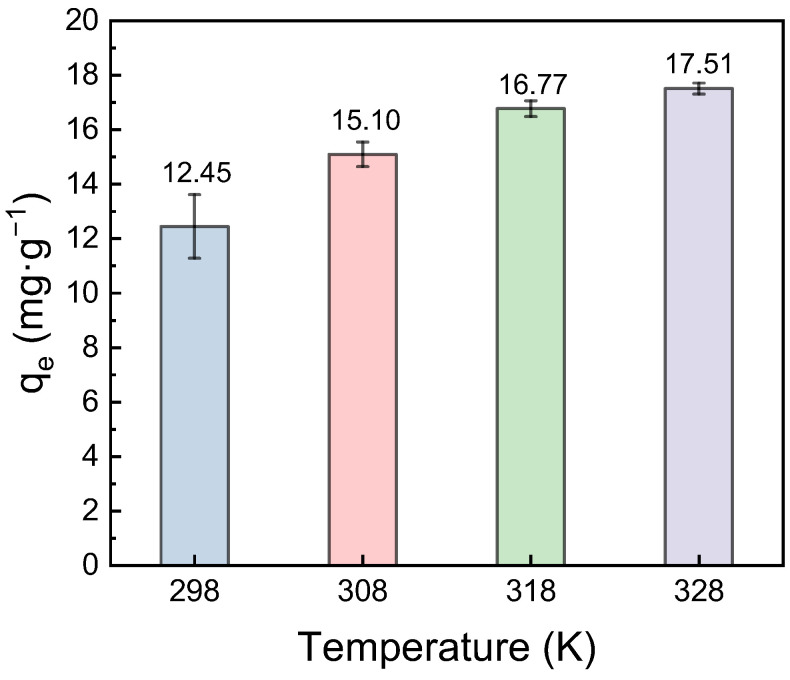
Equilibrium adsorption capacity of Pb(II) on FeOOH@PVDF membranes at different temperatures.

The Pb(II) adsorption capacity of the FeOOH@PVDF membrane was evaluated at four different temperatures (Figure 8). The results showed an increase in adsorption capacity from 12.45 mg·g^−1^ to 17.51 mg·g^−1^ as the temperature rose from 298 K to 328 K. To investigate the thermodynamic characteristics of the adsorption process, the thermodynamic parameters, including the change in Gibbs free energy (ΔG^θ^, kJ·mol^−1^), enthalpy change (ΔH^θ^, kJ·mol^−1^), and entropy change (ΔS^θ^, J·mol^−1^·K^−1^), were calculated using Equations (7) and (8):

(7) $$\Delta G^{\theta }=-RT{\mathrm{lnK}}^{\theta }$$

(8) $$\ln K^{\theta }=\frac{\Delta S^{\theta }}{R}-\frac{\Delta H^{\theta }}{RT}$$

where T is the absolute temperature (K), R is the universal gas constant (8.314 J·mol^−1^·K^−1^), and K^θ^ is the thermodynamic equilibrium constant. The calculated thermodynamic parameter values are presented in Table 4. It can be observed that all ΔG^θ^ values were negative, indicating that the adsorption of Pb(II) ions onto the FeOOH@PVDF membrane was a spontaneous process. Furthermore, the ΔG^θ^ value decreased with increasing temperature, suggesting that higher temperatures favored the adsorption reaction. This finding was consistent with the isothermal adsorption results. Both ΔH^θ^ and ΔS^θ^ were positive, signifying that the adsorption process was endothermic. Consequently, an increase in temperature promoted a higher equilibrium adsorption capacity.

#### 3.2.6. Effect of Ionic Strength

**Figure 9:**
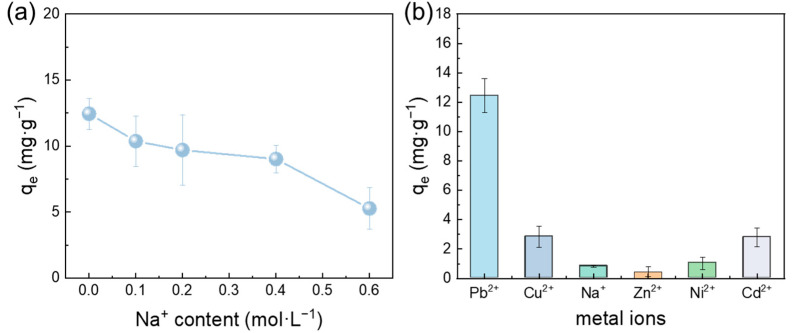
(**a**) Effect of Na+ ion concentration on adsorption capacity; (**b**) equilibrium adsorption capacity of FeOOH@PVDF membrane for different metal ions.

Figure 9a demonstrates that the introduction of Na^+^ ions exerted an inhibitory effect on adsorption. As the Na^+^ concentration in the solution increased, the adsorption capacity for Pb(II) decreased from 12.45 mg·g^−1^ to 5.27 mg·g^−1^. This phenomenon is attributed to competitive adsorption between Na^+^ and Pb(II) ions for the active sites on the membrane surface, which aligns with findings reported in the literature [29]. To investigate the influence of interfering ions on the adsorption of Pb(II) ions by the FeOOH@PVDF membrane, selective adsorption experiments were conducted. The results, presented in Figure 9b, revealed the adsorption capacity sequence as follows: Pb^2+^ (12.45 mg·g^−1^) > Cd^2+^ (2.83 mg·g^−1^) > Cu^2+^ (2.79 mg·g^−1^) > Ni^2+^ (1.03 mg·g^−1^) > Na^+^ (0.81 mg·g^−1^) > Zn^2+^ (0.46 mg·g^−1^). This sequence highlights the high selectivity and efficiency of the resulting membrane for Pb(II) adsorption. The superior selectivity for Pb(II) was primarily due to the strong coordination complexation between Pb^2+^ ions and the abundant hydroxyl (-OH) functional groups on the FeOOH surface. Additionally, Pb^2+^ possessed a higher covalency index. According to coordination chemistry theory, metal ions with larger covalency indices exhibit stronger coordination interactions with ligand groups on the adsorbent.

### 3.3. Membrane Distillation Process for the Heavy-Metal Eemoved Saline Water

**Figure 10:**
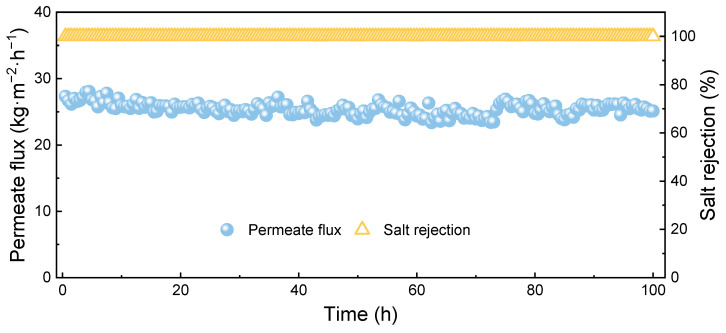
Long-term MD performance of PVDF onmiphobic membrane.

First, the long-term membrane distillation (MD) performance of the omniphobic membrane prepared for our previous work was evaluated for the heavy metal-removed saline water. The liquid entry pressure of the membrane was about 7 bar. The water contact angle approached 180° and thus could not be measured by the static contact angle. As illustrated in Figure 10, the composite membrane exhibited an initial flux of 27.31 kg·m^−2^·h^−1^. Throughout the 100 h continuous MD test, the membrane maintained a high salt rejection rate of 99.99%. Furthermore, it retained a permeate flux of 25.12 kg·m^−2^·h^−1^ after 100 h of operation, demonstrating excellent stability during MD operation.

**Figure 11:**
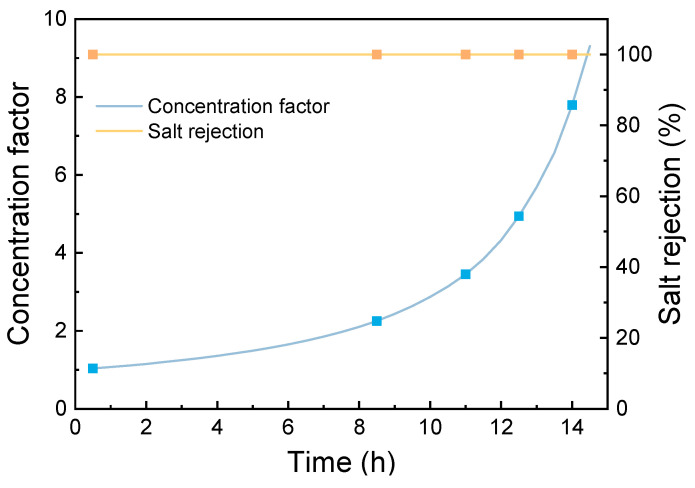
Membrane distillation concentration for the Pb(II)-removed saline water.

Then, the omniphobic membrane was employed to condense the heavy metal-removed saline water as can be seen in Figure 11. The results indicate that the membrane achieved an initial permeate flux of 23.42 kg·m^−2^·h^−1^. After 8.5 h of continuous MD concentration (corresponding to a concentration factor (CF) of 2.2), the membrane still maintained a high permeate flux of 23.23 kg·m^−2^·h^−1^ and a high salt rejection rate of 99.99%. When the operation time was extended to 11 h (CF = 3.4), the permeate flux decreased slightly to 20.17 kg·m^−2^·h^−1^. At 14 h of operation (CF = 7.7), the flux declined to 16.12 kg·m^−2^·h^−1^. Notably, the resulting membrane consistently maintained a high salt rejection rate of 99.99% throughout the entire MD operation, demonstrating stable concentration performance. This excellent concentration performance can be attributed to the membrane’s unique hierarchical micro-nano surface structure, which effectively mitigates concentration polarization and membrane fouling.

Unlike conventional MD processes that face challenges in treating metal-laden saline water, our system effectively separates heavy metals prior to concentration, thereby protecting the MD membrane and improving overall process sustainability. The recovery of heavy metals via adsorption and simultaneous production of high-purity water and concentrated brine offers a circular economy approach, which is rarely achieved in existing studies.

## 4. Conclusions

A novel FeOOH@PVDF adsorption membrane was fabricated in this work and its adsorption performance towards Pb(II) ions was investigated systematically. Based on this, the application potential of an adsorption–membrane distillation coupled process for the integrated treatment of Pb(II)-containing saline water was explored. It was demonstrated that the adsorption process of Pb(II) onto the resulting FeOOH@PVDF membrane conformed more closely to the pseudo-second-order kinetic model and the Langmuir isotherm model, indicating that chemisorption is the dominant mechanism. Thermodynamic analysis revealed that the adsorption process was spontaneous and endothermic (ΔG < 0, ΔH > 0). An increase in temperature favored a rise in the equilibrium adsorption capacity; specifically, within the temperature range of 298 K to 328 K, the adsorption capacity increased from 12.45 mg·g^−1^ to 17.51 mg·g^−1^. In addition, the FeOOH@PVDF membrane exhibited excellent selective adsorption performance, displaying a significantly higher adsorption capacity for Pb(II) compared to other metal ions (Pb^2+^ > Cd^2+^ > Cu^2+^ > Ni^2+^ > Na^+^ > Zn^2+^). MD experiments revealed that the flux was well-maintained at 16.12 kg·m^−2^·h^−1^ even when the concentration factor reached 7.7, highlighting its robust MD concentration capability. The adsorption–MD coupled process successfully partitioned the heavy metal-laden saline water into three distinct streams: heavy metals adsorbed by the membrane, purified water, and concentrated brine. This outcome provides a novel strategy for addressing the pollution caused by high-salinity wastewater containing heavy metals.

## Figures and Tables

**Table 1:** The preparation conditions of different adsorption membranes.

Label	FeOOH (g)	PVDF (g)	DMF (g)
AM1	4.500	3.000	16.50
AM2	4.875	2.625	16.50
AM3	5.250	2.250	16.50
AM4	5.626	1.875	16.50

**Table 2:** Kinetic parameters on the FeOOH@PVDF membrane for Pb(II) in different models.

Pb(Ⅱ) C_0_ (mg·L^−1^)	Pseudo-First Order				Pseudo-Second Order		
	q_e_, exp (mg·g^−1^)	q_e_, cal (mg·g^−1^)	k_1_ (min^−1^)	$R_{1}^{2}$	q_e_, cal (mg·g^−1^)	k_2_ (g·mg^−1^·min^−1^)	$R_{2}^{2}$
25	21.639	11.10	0.0014	0.9517	21.133	0.000711	0.9973

**Table 3:** Adsorption parameters of Langmuir and Freundlich isotherm models.

Langmuir			Freundlich		
*Q_m_* (mg·g^−1^)	*K_L_* (L·mg^−1^)	$R_{L}^{2}$	*K_F_* [(mg·g^−1^) (L·mg^−1^)^(1/n)^]	*n*	$R_{F}^{2}$
29.68	0.1392	0.9765	5.61	2.21	0.7448

**Table 4:** Thermodynamic data for adsorption of Pb(II) ions on FeOOH@PVDF membrane.

Temperature (K)	Δ*G^θ^* (kJ·mol^−1^)	Δ*H^θ^* (kJ·mol^−1^)	Δ*S^θ^* (kJ·mol^−1^·K^−1^)
298	−2.70	34.36	0.1246
308	−4.00		
318	−5.44		
328	−6.35		

## Supplementary Materials

The following supporting information can be downloaded at: https://www.mdpi.com/article/10.3390/membranes15090271/s1, Table S1: the properties of the adsorption membrane (AM3).
