# Supplementary material for: Synergistic Adsorption–Membrane Distillation for Heavy Metal Extraction and Water Reclamation from Saline Waste Streams
Source: Membranes (Basel). 2025 Sep 8;15(9):271. doi: 10.3390/membranes15090271 (PMC12471472; doi:10.3390/membranes15090271)
Supplement: Supplementary file 1 [file membranes-15-00271-s001.zip › membranes-3841264-supplementary.pdf]

Synergistic Adsorption–Membrane Distillation for Heavy Metal Extraction and Water Reclamation from Saline Waste Streams

Jie Xu [1], Jinxin Liu [1], Mei-Ling Liu [2,3], Guangze Nie [1,*] and Dong Zou [1,2,*]

[1] School of Environmental Science and Engineering, Nanjing Tech University, Nanjing 211816, China
[2] NJTECH University Suzhou Future Membrane Technology Innovation Center, Suzhou 215333, China
[3] State Key Laboratory of Materials-Oriented Chemical Engineering, College of Chemical Engineering, Nanjing Tech University, Nanjing 211816, China
* Correspondence: gznie@njtech.edu.cn (G.N.); zoudong@njtech.edu.cn (D.Z.)

Table S1. The properties of the adsorption membrane (AM3).

Porosity	Pore size	thickness	Size
80%	252 nm	150 μm	*

Effect of pH on Adsorption Performance:

A predetermined amount of $Pb(NO_3)_2$ was accurately weighed and dissolved to prepare a standard stock solution of Pb(II). An appropriate volume of the stock solution was diluted to 10.0 $mg \cdot L^{-1}$, and the pH was adjusted within the range of 2.0-7.0 using NaOH and HCl solutions to prepare 20.0 mL of simulated heavy metal-containing wastewater containing Pb(II). Then, 10.0 mg of the adsorptive membrane was added to the solution. The mixture was placed in a constant-temperature shaker at 25°C and agitated at 200 rpm for 24 h to ensure adsorption equilibrium was reached. After the reaction, the supernatant was extracted using a syringe and filtered through a 0.22 μm aqueous filter membrane. The equilibrium concentration of Pb(II) was measured, and the adsorption capacity was calculated.

Adsorption Performance of Different Membranes:

A Pb(II) solution with a concentration of 10.0 $mg \cdot L^{-1}$ was prepared, and 20.0 mL of the solution was transferred into a centrifuge tube. The pH was adjusted to 6.0. Then, 10.0 mg of the adsorptive membrane was added to the solution. The mixture was shaken in a constant-temperature shaker at 25°C and 200 rpm for 24 h. After adsorption, the supernatant was collected, filtered through a 0.22 μm aqueous filter membrane, and the equilibrium concentration of Pb(II) was determined to calculate the adsorption capacity.

Effect of Contact Time:

A simulated wastewater solution was prepared with 500.0 mL of Pb(II) at a concentration of 25.0 $mg \cdot L^{-1}$. The pH was adjusted to 6.0, and 250.0 mg of the adsorptive membrane was added. The adsorption experiment was conducted under mechanical stirring at a constant speed at room temperature. Samples were taken at different time intervals, and the supernatant was filtered through a 0.22 μm membrane filter. The Pb(II) concentration was measured, and the adsorption capacity at each time point was calculated to investigate the adsorption kinetics.

Effect of Pb(II) Concentration:

Simulated wastewater solutions with Pb(II) concentrations of 5, 10, 15, 25, 40, and 50 $mg \cdot L^{-1}$ were prepared in 20.0 mL volumes. The pH was adjusted to 6.0, and 10.0 mg of the adsorptive membrane was added to each solution. The mixtures were shaken at a constant temperature and 200 rpm for 24 h until adsorption equilibrium was achieved. The supernatant was then extracted and filtered, and the Pb(II) concentration was measured to determine the equilibrium adsorption capacity.

Effect of Reaction Temperature:

A simulated heavy metal wastewater solution containing Pb(II) at 10.0 $mg \cdot L^{-1}$ was prepared. Then, 20.0 mL of the solution was placed in a centrifuge tube, and the pH was adjusted to 6.0. Subsequently, 10.0 mg of the adsorptive membrane was added. The constant-temperature shaker was set to 25, 35, 45, and 55°C, respectively, and the mixtures were shaken at 200 rpm for 24 h. After adsorption equilibrium was reached, the supernatant was extracted, filtered, and the Pb(II)

concentration was measured to calculate the equilibrium adsorption capacity, thereby evaluating the effect of temperature on adsorption performance.

Effect of Ionic Strength:

To further investigate the practical application potential of the adsorptive membrane in heavy metal-laden saline water, the influence of different Na^+ concentrations on adsorption performance was studied. Specific amounts of sodium nitrate were weighed and dissolved in a 10.0 $mg \cdot L^{-1}$ Pb(II) solution to prepare lead-containing solutions with Na^+ concentrations of 0.1, 0.2, 0.4, and 0.6 $mol \cdot L^{-1}$. Then, 20.0 mL of each solution was taken, the pH was adjusted to 6.0, and 10.0 mg of the adsorptive membrane was added. The mixtures were shaken in a constant-temperature shaker at 25°C and 200 rpm for 24 h. After adsorption equilibrium, the supernatant was extracted and filtered, and the Pb(II) concentration was measured to calculate the equilibrium adsorption capacity and analyze the effect of ionic strength.

Selective Adsorption:

To evaluate the selective adsorption capability of the membrane for Pb(II), appropriate volumes of standard stock solutions of Pb(II), Cu(II), Zn(II), Cd(II), Ni(II), and Na(I) were taken to prepare 20.0 mL metal ion solutions each at a concentration of 10.0 $mg \cdot L^{-1}$. Then, 10.0 mg of the adsorptive membrane was added to each solution. The mixtures were shaken in a constant-temperature shaker at 25°C and 200 rpm for 24 h. After adsorption equilibrium, the supernatant was collected, filtered, and the equilibrium concentrations of the metal ions were measured.
